# Syndromic management of sexually transmitted infections among female sex workers in Lomé (Togo), 2023

**DOI:** 10.1371/journal.pone.0337100

**Published:** 2025-11-17

**Authors:** Oumarou I. Wone Adama, Iman Frédéric Youa, Alexandra Bitty-Anderson, Arnold Junior Sadio, Rogatien Comlan Atoun, Yao Rodion Konu, Hezouwe Tchade, Martin Kouame Tchankoni, Kokou Herbert Gounon, Kparakate Bouboune Kota-Mamah, Abissouwessim Egbare Tchade, Godonou Amivi Mawussi, Fiali Ayawa Lack, Fifonsi Adjidossi Gbeasor-Komlavi, Anoumou Claver Dagnra, Didier Koumavi Ekouevi

**Affiliations:** 1 African Centre for Research in Epidemiology and Public Health (CARESP), Lomé, Togo; 2 University of Bordeaux, National Institute of Medical Research (Inserm) UMR 1219, Institute of Research for Development (IRD) EMR 271, Bordeaux Population Health Centre, Bordeaux, France; 3 Faculty of Health Sciences, Department of Public Health, University of Lomé, Lomé, Togo; 4 Faculty of Health Sciences, Laboratory of Molecular Biology and Immunology, University of Lomé, Lomé, Togo; University of Zimbabwe Faculty of Medicine: University of Zimbabwe College of Health Sciences, ZIMBABWE

## Abstract

**Introduction:**

In Togo, the syndromic approach is used for the diagnosis and management of sexually transmitted infections (STIs). The aim of this study was to evaluate the syndromic approach for diagnosis of STIs among female sex workers (FSW) in Lomé, Togo.

**Methods:**

A cross-sectional study was carried out from September to October 2023 among FSW in Lomé (Togo). FSW aged 18 years and above were included. A gynecological examination was performed for syndromic diagnosis, and the Xpert® CT/NG were used to screen vaginal swabs for *Chlamydia trachomatis* (CT) and *Neisseria gonorrhoeae* (NG). The performance (predictive values) of the syndromic approach to STI diagnosis was evaluated using the Xpert® CT/NG test as the gold standard.

**Results:**

A total of 357 FSW were recruited. The median age of FSW was 32 years (IQR: [26–40 years]) and 8.2% had attained a higher level of education. The prevalence of syndromic STI among FSW was 33.3%. Vaginal swabs were positive for CT (8.4%) and NG (8.7%), with a prevalence of bacterial STIs (CT and/or NG) of 14.3%. The syndromic approach to STI diagnosis demonstrated a positive predictive value of 24.3%.

**Conclusion:**

The prevalence of STIs is relatively high among FSW in Lomé. According to this study, the diagnosis of STIs using the syndromic approach has limited relevance. National STI screening and management policies urgently need to be rethought, incorporating recent technological advances.

## Introduction

Sexually transmitted infections (STIs) represent a considerable burden on health and the global economy with approximately 1 million new curable infections per day [1]. The main STIs are syphilis (7.1 million), gonorrhea (82 million), chlamydia (129 million) and trichomoniasis (156 million). The presence of these STIs increases the risk of contracting and transmitting the Human Immunodeficiency Virus (HIV) [1]. In 2020, the World Health Organization (WHO) estimated that there were 374 million new cases of these four STIs among people aged 15–49 worldwide.

In sub-Saharan Africa, STI prevalence data show great variability but show a higher burden among female sex workers (FSW) due to the nature of their profession, their vulnerability to violence, and their limited access to health services [2,3].

In most countries in sub-Saharan Africa and low- and middle-income countries (LMICs), syndromic management of STIs is recommended, and the 2021 WHO syndromic management algorithms remain the standard of care for STI prevention and control [4]. These guidelines specifically target STI syndromes such as urethral discharge, vaginal discharge or persistent vaginal discharge, anorectal infections, genital ulcerations, and lower abdominal pain [4]. Establishing an etiological diagnosis of STIs, while ideal, is very often difficult or even impossible in these contexts for several reasons, including the lack of established laboratories or competent human resources [5,6]. However, several studies question this approach, citing the low positive predictive value (PPV) for STI diagnosis, as well as the potential danger of overtreatment and antimicrobial resistance [7,8]. Indeed, studies have reported an increasing proportion of antimicrobial resistance in *N. gonorrhoeae* [9,10]. This is the reason why the WHO has recommended that when this approach is used, it should be supplemented by etiological studies at regular intervals. These etiological studies make it possible to identify the main germs responsible for STIs in the population and to verify the relevance and effectiveness of the antibiotic therapy regimens proposed in the syndromic approach [11].

In Togo, in the routine of peripheral centers, the treatment of STIs is still based on the identification of signs and symptoms using algorithms developed by the WHO since 1991 [12]. In 2017, estimates made based on biological tests indicated among FSW in Togo, STI prevalences of 4.2% for *Neisseria gonorrhoeae* (NG), 6.1% in favor *Chlamydia trachomatis* (CT), 5.5% for *Mycoplasma genitalium* (MG) and 6.5% for *Trichomonas vaginalis* (TV) [13,14]. However, no studies have compared the two approaches (syndromic and biological). The main objective of this study was to evaluate the syndromic approach in STI screening among FSW in Lomé (Togo) in 2023.

## Methods

### Outline and framework of study

This was a cross-sectional study carried out in Lomé from September to October 2023. Lomé is the political capital of Togo and is a cosmopolitan city that alone accounts for nearly half of the population of FSW in Togo [15].

The study was conducted on the clinical site of the NGO FAMME (Forces in Action for the Well-Being of Mother and Child). This NGO’s mission is to defend, promote the rights and interests and improve the health and socio-economic well-being of the most vulnerable women and their children. The NGO FAMME has a health center and is also an HIV care site.

### Study population and selection of participants

The selection of FSW was carried out through a so-called snowball recruitment method, in collaboration with other local organizations involved with FSW. They were recruited at the level of the “hot spots” identified by the leaders of the FSW and the peer educators. The inclusion criteria were: i) women aged 18 years or older, ii) self-identifying as FSW, and iii) who freely consented to participate in the study. Those who did not meet the conditions set for the vaginal swab, i.e.,: i) not be in the menstrual period or ii) have benefited from local or systemic antibiotic therapy in the three days prior to the survey, were not included in this study.

### Sample size

To determine the sample size required, we used the formula used for the design of diagnostic accuracy studies as described by Akoglu [16]. For an expected prevalence of bacterial STIs, estimated at 30.0%, a sensitivity of the syndromic approach of 55% according to a study in India [17], a first-kind error of 5% and a 95% confidence level, at least 320 FSW should be included.

### Data collection

#### Behavioral and clinical data.

Information on sociodemographic characteristics, sexual practices, history and current symptoms of STIs was obtained using a digitized questionnaire, administered face-to-face by doctors trained in the survey procedures. For those who did not speak French, the questionnaire was administered in English or local languages to facilitate exchanges. A gynecological examination for the collection of clinical data was then conducted in all FSW.

#### Biological data and laboratory analysis.

Following this examination, HIV serology and cervicovaginal swabs were performed. These samples were analyzed at the National Reference Tuberculosis Laboratory (NRL-TB) of the Sylvanus Olympio University Hospital Center (CHU-SO) using the Xpert^®^ CT/NG test (Cepheid^®^, Sunnyvale, CA, USA) for the search for CT and NG.

### Operational definitions

We defined FSW as carriers of a syndromic STI as those with a symptom of STI (genital discharge, genital ulceration, pruritus, pain when urinating) at the time of the survey. All FSW who tested positive for CT or NG were classified as carriers of a bacterial STI, whether symptomatic or not.

Syndromic diagnosis of STIs based on the WHO STI management algorithm, was the evaluated method and (biological) diagnosis by Xpert® test was the reference method considered to be *Gold standard*.

For the detection of CT on vaginal swabs, the Xpert^®^ has a sensitivity and specificity of 99.5 (97.3–100) and 99.1 (98.8–99.4) respectively regardless of the presence of symptoms. For the detection of NG on vaginal swabs, the Xpert® test has a sensitivity and specificity of 100% (93.2–100) and 99.9% (99.8–100) respectively regardless of the presence of symptoms. The positive predictive value of the Xpert® test for CT and NG is 86.6% (81.5–90.7) and 94.5% (84.9–98.9), respectively, regardless of the presence of symptoms [18].

The level of agreement was assessed using the Cohen Kappa coefficient (κ) between −1 and +1, and interpreted as follows: values ≤ 0 indicate no agreement and 0.01–0.20 zero to slight agreement, 0.21–0.40 low agreement, 0.41–0.60 moderate agreement, 0.61–0.80 substantial agreement, and 0.81–1.00 almost perfect agreement [19].

### Statistical analysis

Descriptive statistics were produced, and the results were presented with tables of numbers and proportions for the qualitative variables. Quantitative variables were presented as a median with their interquartile range (IQR).

To assess the agreement between the syndromic diagnosis of STIs and the biological diagnosis, in addition to the percentage of agreement, a Kappa coefficient was calculated. The performance of syndromic diagnosis of STIs was measured through the calculation of its intrinsic (sensitivity and specificity) and extrinsic (positive and negative predictive values) parameters. All analyses were performed using R© software version 4.3.2.

### Ethical and regulatory aspects

This study received a favorable opinion from the Bioethics Committee for Health Research (CBRS) of the Ministry of Health and Public Hygiene of Togo. FSW participating in the study were informed of the progress of the study by the investigators. Informed, written consent was obtained prior to inclusion in the study. FSW with STI symptoms during the survey were all treated according to national recommendations.

## Results

### Sociodemographic characteristics of sex workers (FSW)

A total of 357 FSW were included. The median age was 32 years (IQR: [26–40 years]) and 8.2% had attained a higher level of education. More than half of FSW, 52.9%, had a regular partner and 11.3% lived in brothels (**Table 1**).

**Table 1:** Distribution of FSW by socio-demographic characteristics, Lomé, 2023, N = 357.

Characteristics	N	Proportion (%)
**Age (years)**		
Minimum-Maximum	18-68	
Median (IQR)	32 (26-40)	
**Age Groups (years)**		
18 - 29	141	39.5
30 - 39	121	33.9
≥ 40	95	26.6
**Level of education**		
Non-schoolchildren	53	14.8
Primary	125	35.0
Secondary	150	42.0
Upper	29	8.2
**Type of living space**		
Collective	225	63.0
Individual	92	25.7
Brothel	40	11.3
**Having a regular customer**		
No	120	33.6
Yes	237	66.4
Having a partner*		
No	168	47.1
Yes	189	52.9

*Boyfriend, husband, IQR: interquartile range.

### Sexual history of FSW

The median age at first sexual intercourse was 18 years (IQR: [16–20 years]) and the median age at first paid sex was 24 years (IQC: [20–30 years]). Sex work was considered the main source of income for almost half of FSW (48.2%). The **Table 2** presents the sexual history of FSW in Lomé in 2023.

**Table 2:** Sexual history of FSW, Lomé, 2023, N = 357.

Characteristics	N	Proportion (%)
**Age at first sexual intercourse (years)**		
Minimum-Maximum	10-30	
Median (IQR)	18 (16-20)	
**Age at first paid sexual intercourse (years)**		
Minimum-Maximum	14-51	
Median (IQR)	24 (20-30)	
**Sex work as a main activity**		
No	185	51.8
Yes	172	48.2
**Number of sexual partners in the week prior to the study**		
None	15	4.2
1 - 4	180	50.4
≥ 5	162	45.4

IQR: interquartile range.

### STIs among FSW

#### STI symptoms.

**Fig 1:**
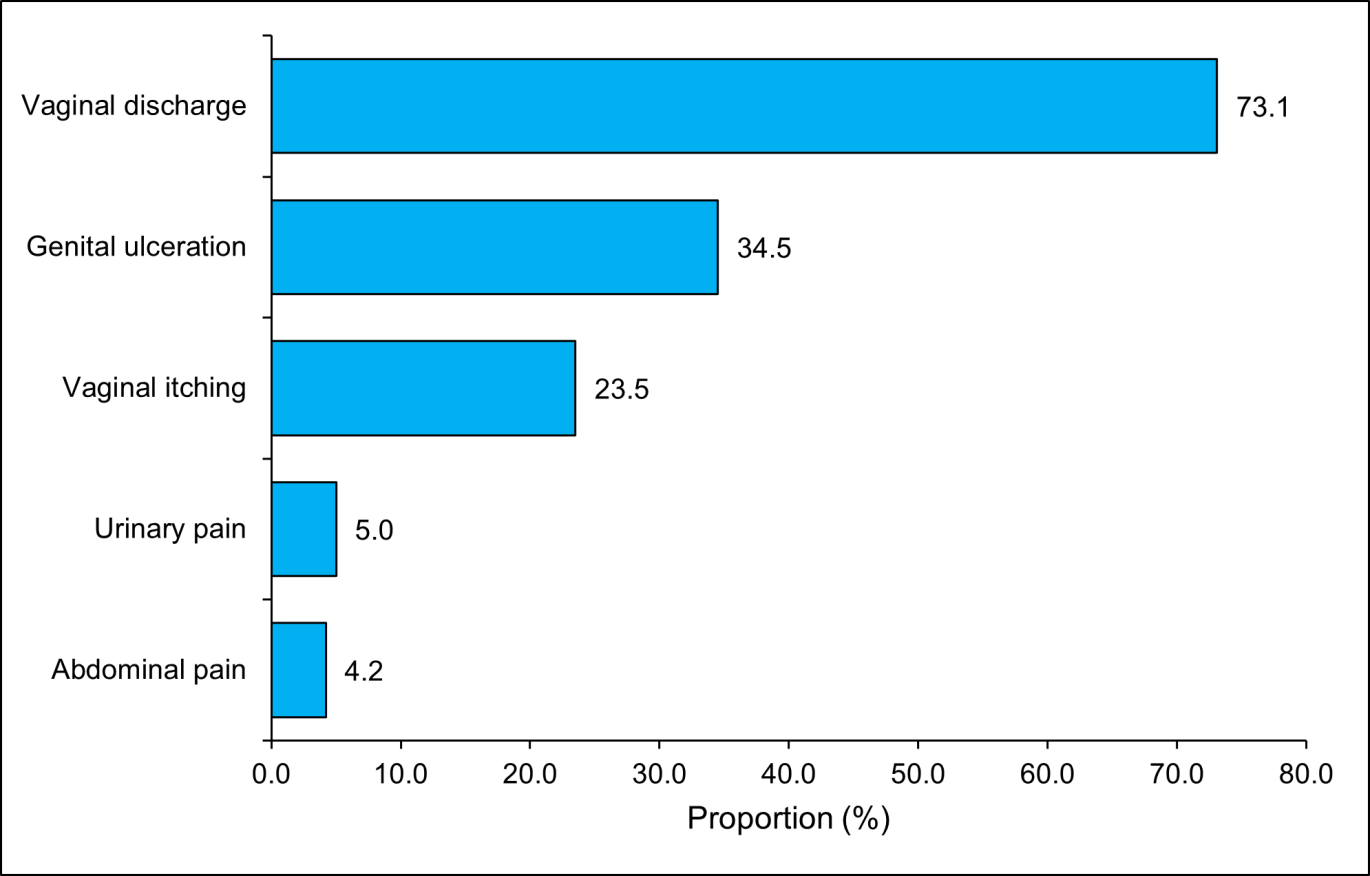
STI symptoms in FSW, Lomé, 2023, N = 119.

STI symptoms were identified by doctors in 119 FSW, which corresponds to a proportion of 33.3%. The most frequent symptoms were vaginal discharge in 73.1% of cases, followed by genital ulcers (34.5%) and vaginal itching (23.5%). Conversely, voiding and abdominal pain were the least reported (**Fig 1**).

#### STIs suspected according to the syndromic approach using the management algorithm.

**Fig 2:**
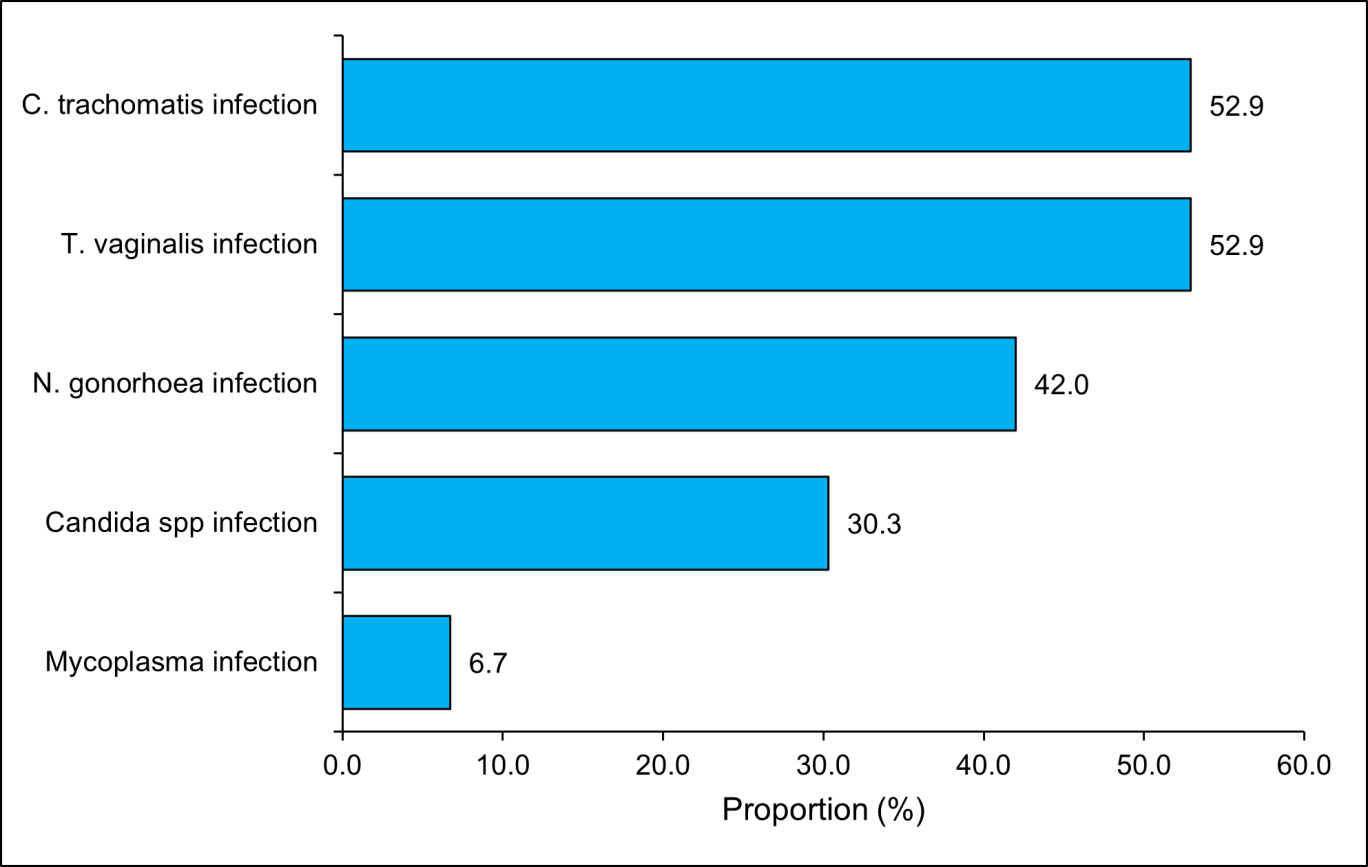
STIs suspected according to the syndromic approach using the management algorithm in FSW, Lomé, 2023, N = 119.

Referring to the national algorithm for the management of STIs in Togo, the STIs suspected by the doctors in the study were *T. vaginalis* and *C. trachomatis* (52.9%) followed by *N. gonorrhoeae* (42,0%) (**Fig 2**).

### Laboratory results (biological diagnosis)

#### Prevalence of STIs.

Based on the biomolecular results, the estimated prevalence of infection with *Chlamydia trachomatis* and *Neisseria gonorrhoeae* was 8.4% and 8.7%, respectively. Overall, 14.3% of the FSW surveyed were carriers of a bacterial STI (CT or NG). In addition, the prevalence of HIV infection has been estimated at 15.4%.

#### Concordance between the syndromic approach and the biological diagnosis of sexually transmitted infections.

A total of 247 concordant results (69.2%) were recorded between the two diagnostic approaches, i.e., 30 positive concordant results and 217 negative concordant results. The **Table 3** presents the cross-reference between the results of the two diagnostic approaches.

**Table 3:** Agreement between syndromic diagnosis and laboratory diagnosis.

	Laboratory Diagnosis		
	CT/NG +	CT/NG -	Total
**Syndromic approach**			
CT or NG +	18 **(a)**	56 **(b)**	74 **(e)**
CT or NG -	33 **(c)**	250 **(d)**	283 **(f)**
Total	51 **(g)**	306 **(h)**	357 **(i)**

STI: Sexually transmitted infection diagnosed in the laboratory.

STI syndrome: sexually transmitted infection diagnosed based on the syndromic approach.

The Kappa coefficient was calculated at 0.14; 95% CI [0.027–0.26], reflecting a low level of agreement between the syndromic approach and the biological diagnosis. Regarding diagnostic performance, the syndromic approach had a sensitivity of 35.3% and a positive predictive value of 24.3%. The **Table 4** presents the parameters of concordance between syndromic diagnosis and biological diagnosis of STIs.

**Table 4:** Performance parameters of syndromic diagnosis and biological diagnosis of STIs.

Parameters	Value	95%CI
Sensitivity (%)	35.3	22.1–48.4
Specificity (%)	81.7	77.4–86.0
Positive predictive value (%)	24.3	14.5–34.1
Negative predictive value (%)	88.3	84.6–92.1
Positive likelihood ratio	1.92	–
Negative likelihood ratio	0.79	–

95%CI: 95% Confidence Interval.

Sensitivity: (a/g)*100.

Specificity: (d/h)*100.

Positive predictive value: (a/e)*100.

Negative predictive value: (c/f)*100.

Positive likelihood ratio: ***Sensitivity/(1- Specificity)*.**

Negative likelihood ratio: ***(1- Sensitivity)/ Specificity*.**

## Discussion

The syndromic approach to the management of STIs is mainly applied in Togo [20]. WHO recommends regular re-evaluation of this approach with the aim of limiting the risk of antibiotic resistance emerging. The present study documented syndromic STIs and made a biological diagnosis in FSW. These conditions made it possible to measure the intrinsic (sensitivity/specificity) and extrinsic (predictive values) parameters of the syndromic approach and to evaluate its effectiveness as a diagnostic method.

Sensitivity and specificity are relevant statistical parameters for evaluating the performance of a diagnostic test [21]. However, in practice, rather than knowing the proportion of sick patients who will test positive (sensitivity) or non-sick patients who will test negative (specificity), it is often more useful to predict whether a person will actually have the disease based on a positive or negative test result (positive/negative predictive values) [22,23]. In this sense, the positive predictive value is the most useful indicator for clinical decision-making [22]. The usefulness of this indicator is that it allows us to know how many of those who will test positive are actually sick [22]. Furthermore, the prevalence of the disease has a strong influence on the positive and negative predictive values: a positive result is more reliable when the disease is common, while a negative result is more reliable when the disease is rare [23]. In the present study, the syndromic approach had a sensitivity of 35.3% and a positive predictive value of 24.3%. In other words, among people who were truly infected with an STI, the syndromic approach allowed a diagnosis in 35.3% of cases, and only a quarter of people declared to be carriers of an STI by the syndromic approach were infected with one of the STIs studied. These results suggest that the syndromic approach, although widely used in Togo and sub-Saharan Africa, has limitations in the diagnosis of STIs. Other studies in similar settings have also reported the limitations of the syndromic approach to STI screening and management. Indeed, a meta-analysis carried out by Zemouri in 2016 over a 15-year period in low- and middle-income countries reported a low diagnostic performance of the syndromic approach and a high proportion of missed or overtreated [24]. In another study of 276 women in Senegal in 2018, the use of the syndromic approach resulted in adequate management for only 51% of women with *Trichomonas vaginalis*(TV) and *Gardnerella vaginalis*(GV) and for 54% of those with *Chlamydia trachomatis*(CT) and *Neisseria gonorrhoeae* (NG) [25].

In addition, this study documented less common but clinically relevant indicators. These are diagnostic or *likelihood ratios*. The *Positive likelihood ratios (LR*+) indicates how more common positive test result is in people with the disease compared to those without. Such a ratio equal to 1 is not informative because it would mean as many chances of having a positive test in both sick and non-sick people [26]. The data from this study made it possible to calculate this ratio which was 1.92. In other words, it is 2 times more likely that truly infected FSW will show signs suggestive of STIs than those who are not. It also suggests that clinicians should pay particular attention to any clinical manifestation suggestive of an STI by prescribing additional laboratory tests and ensuring close monitoring of symptomatology.

One of the main criticisms of the syndromic approach is its inability to detect asymptomatic cases, which are increasingly recurrent in key populations [27]. It has been reported that bacterial STIs in women in general are mostly asymptomatic with only 6–17% and 14–35% of women becoming symptomatic, for CT and NG, respectively [28]. A recent study of sex workers in Zimbabwe to evaluate the Ministry of Health and Child Care’s syndromic case management algorithm for vaginal discharge in 2018 found that a high proportion were asymptomatic, with 41.2% of cases being gonorrhoea, 51.7% chlamydia, and 62.8% trichomoniasis. [29]. A study of high-risk women in South Africa, based on national recommendations, revealed that the syndromic approach failed to identify approximately 88% of cases of sexually transmitted infections (STIs) that were diagnosed through laboratory testing [8]. Similar results were reported in Kisumu, Kenya with 846 participants. In this study, the agreement between syndromic and etiological diagnoses of STIs was low (κ = 0.09). In this study, the syndromic approach, based on Kenyan national guidelines aligned with WHO recommendations for STI management, proved to be insensitive, identifying only 10.4% of participants as positive for an STI based on clinical symptoms, compared to 32.2% confirmed by laboratory tests. Herpes simplex virus (HSV)-2 infection has been severely underdiagnosed, while CT or NG infections have been over diagnosed using the syndromic approach [30]. These results highlight an important challenge in the effective management of STIs using the syndromic approach. However, for low- and middle-income countries, the use of the etiological approach for STI diagnosis is not sustainable. One proposed solution would be to offer presumptive periodic treatments based on a risk assessment [29].

The syndromic approach, which is based on subjective judgment, often leads to misdiagnosis [27]. Physiological losses in women can sometimes be misinterpreted as pathological. A study conducted in India in 2000 among FSW in the red-light district of Surat established that the syndromic approach based on vaginal discharge had a low specificity (50–55%) and a low positive predictive value, missing 30–40% of CT and NG cases and leading to treatment in up to 90% of cases without infection [17]. Similar results were reported in women attending outlying government clinics in Delhi, India in 2004, where the reported specificity of the syndromic approach using vaginal discharge as a symptom was only 37.5% [31]. Such a disparity between syndromic and etiological diagnosis was also reported in Sudan in 2016 where the syndromic approach showed that among participants with vaginal discharge, only 14.7% had a true STI [32]. In Zimbabwe and Senegal, in 2018 and 2019 respectively, 27% to 49% of women with vaginal discharge were not diagnosed positive for an STI [7,33]. Given that 73.1% of FSW had vaginal discharge in the present study, it is possible that some of these are physiological discharge that was misinterpreted as pathological by the investigating physicians. These results highlight the difficulty in distinguishing normal discharge from signs of infection in the syndromic approach, highlighting the need for extensive training of practitioners and the importance of integrating complementary methods to improve diagnostic accuracy. While the syndromic approach remains the most practical method in the management of STIs in developing countries, it is important that capacity building and continuous training of practitioners be a priority, particularly for clinicians involved in the care of the most vulnerable populations. In addition, investing in research for the availability of low-cost, simple and rapid point-of-care STI diagnostic tests should be a public health issue.

This study is one of the few to have evaluated the syndromic approach for the diagnosis of STIs in FSW in Togo. This study has some limitations, in particular the non-performance of throat and anal samples for a complete exploration of these STIs. Another limitation is that the present study does not take into account the biological diagnosis of other STIs such as *Trichomonas vaginalis*, *Mycoplasma genitallium* and *Treponema pallidum* which were found in 6.5%, 5.5% and 0.8% of FSW cases in Togo in 2017 respectively [14,34]. In other words, it is possible that a participant had symptoms of STIs and had a negative test (CT/NG). This could partly explain the discrepancy in the results observed between syndromic (33.3%) and etiological (14.3%) diagnosis of STIs. To minimize this bias, one adjustment strategy would be to restrict concordance analyses to FSW with signs suggesting CT and NG infections. The results should therefore be interpreted with caution and should be considered as a first exploratory approach.

However, the results presented suggest the need for a large-scale evaluation and possibly a readjustment of the syndromic approach currently used in Togo. The reflection must also take into account technological developments and be oriented towards the acquisition of rapid tests for STIs [35].

## Conclusion

The present study suggests that diagnosis based on the syndromic approach is of little relevance. Also, the prevalence of bacterial STIs (CT or NG) remains relatively high among FSW in Lomé. There is an urgent need to plan a more comprehensive evaluation of the syndromic approach to STI screening and management and to readjust it based on the available data in FSW. The development of future STI management policies should incorporate recent technological advances such as rapid diagnostic tests that consider several pathogens at the same time.

## Abbreviations

95%CI: 95% Confidence Interval
CBRS: Bioethics Committee for Health Research
CHU-SO: Sylvanus Olympio University Hospital Center
CT: Chlamydia trachomatis
FSW: Female sex workers
IQR: Interquartile range
LMICs: Low- and middle-income countries
NG: Neisseria gonorrhoeae
NLR-TB: National Reference Tuberculosis Laboratory
STI: sexually transmitted infections
WHO: World Health Organization
